# Genomic Characterization of Clinical *Acinetobacter baumannii* Isolates Obtained from COVID-19 Patients in Russia

**DOI:** 10.3390/antibiotics11030346

**Published:** 2022-03-06

**Authors:** Andrey Shelenkov, Yulia Mikhaylova, Lyudmila Petrova, Irina Gaidukova, Mikhail Zamyatin, Vasiliy Akimkin

**Affiliations:** 1 Central Research Institute of Epidemiology, Novogireevskaya Str., 3a, 111123 Moscow, Russia; mihailova@cmd.su (Y.M.); vgakimkin@yandex.ru (V.A.); 2 National Medical and Surgical Center named after N.I. Pirogov, Nizhnyaya Pervomayskaya Str., 70, 105203 Moscow, Russia; lutix85@yandex.ru (L.P.); 0609irina@rambler.ru (I.G.); mnz1@yandex.ru (M.Z.)

**Keywords:** *Acinetobacter baumannii*, genomic epidemiology, whole genome sequencing, healthcare-associated infections, antibiotic resistance, COVID-19

## Abstract

The coronavirus disease 2019 (COVID-19) pandemic has already affected all realms of public healthcare and, in particular, has led to increasing use of various antibiotics to treat possible bacterial coinfections even in cases for which such infections were not confirmed clinically. This could lead to an increase in the fraction and severity of multidrug-resistant bacterial isolates in healthcare facilities, especially in intensive care units (ICU). However, detailed epidemiological investigations, possibly including whole genome sequencing (WGS), are required to confirm the increase in antibiotic resistance and changes, if any, in the population and clonal structures of bacterial pathogens. In this study, we performed a comprehensive genomic and phenotypic characterization of selected multidrug-resistant *A. baumannii* isolates obtained from the patients of a dedicated COVID-19 ICU in Moscow, Russia. Hybrid short- and long-read sequencing allowed us to obtain complete profiles of genomic antimicrobial resistance and virulence determinants, as well as to reveal the plasmid structure. We demonstrated the genomic similarity in terms of cgMLST profiles of the isolates studied with a clone previously identified in the same facility. We believe that the data provided will contribute to better understanding the changes imposed by the COVID-19 pandemic on the population structure and the antimicrobial resistance of bacterial pathogens in healthcare facilities.

## 1. Introduction

The coronavirus disease 2019 (COVID-19) pandemic affected virtually all fields of medicine, and the treatment of healthcare-associated infections is not an exception. The too-frequent and inappropriate use of antibiotics is a major concern; for example, recent meta-analysis has shown that more than 70% of hospitalized COVID-19 patients received antibiotics, while only about 9% demonstrated superimposed bacterial or fungal coinfections [1]. According to another investigation, in 96% of cases the antibiotic treat-ment was prescribed before a bacterial infection was confirmed [2]. The effects of such overuse are currently hard to estimate due to limited data available [3], but the worldwide increase in resistance is a very likely scenario. However, further investigations and continuous surveillance are required to draw reliable conclusions regarding the influence of COVID-19 on bacterial resistance to antimicrobial drugs in clinical settings. An important step in this direction seems to be the monitoring of the bacterial coinfections of COVID-19 patients admitted to dedicated intensive care units (ICU) and a comparison of such infections with those found pre-COVID-19, especially, on the level of genomic resistance determinants for particular pathogens.

The pathogens belonging to the ESKAPE group (*Enterococcus faecium*, *Staphylococcus aureus*, *Klebsiella pneumoniae*, *Acinetobacter baumannii*, *Pseudomonas aeruginosa* and *Enterobacter* spp.) are the major causes of antibiotic-resistant infections worldwide [4], and are especially dangerous in hospitals due to their ability to rapidly acquire and maintain resistance to a broad spectrum of antimicrobial drugs [5]. In recent years, *A. baumannii* became the third most common pathogen (being inferior only to *K. pneumoniae* and *P. aeruginosa*) causing infections in clinical settings in Russia according to AMRmap [6] (https://amrmap.ru/, accessed on 10 January 2022). This pathogen accounts for about 15% of the clinical infection cases in Russia in 2018–2020, and its resistance to carbapenems reaches 80% [6]. The ability of *A. baumannii* to easily survive and transfer in the hospital environment via its attachment to various biotic and abiotic surfaces makes its treatment and eradication even more complicated [7].

In the current preliminary study, we sequenced the genomes of four representative multidrug-resistant (MDR) *A. baumannii* isolates obtained from the patients at a dedicated COVID-19 ICU in a multidisciplinary medical center located in Moscow, Russia during the first wave of the pandemic. Two of these isolates were subjected to long-read sequencing in order to obtain reliable plasmid structures and facilitate a whole genome comparison. Phenotypic and genomic antibiotic resistance were characterized and the virulence factors and plasmids were described. The isolates obtained were compared with each other, as well as with different *A. baumannii* isolates previously obtained in this medical center [8] and the isolates of the same sequence type (ST) available in public databases.

## 2. Results

The results of antibiotic susceptibility testing and the metadata for the isolates studied are provided in Table 1.

All isolates were resistant to the antibiotics from a short panel. Furthermore, CriePir331 and CriePir332 were subjected to both short- and long-read sequencing, while two other isolates were sequenced using the short-read sequencer only. The hybrid assemblies of short- and long-read genome sequences of CriePir331 and CriePir332 included the long chromosomal contigs of 4,022,563 and 4,016,612 bp, respectively. These two isolates also contained an additional shorter contig of plasmid origin with the same lengths of 11,194 bp. The CriePir333 and CriePir345 contained 80 and 86 contigs with a length greater than 500 bp, respectively.

Annotations of the genomic antibiotic resistance determinants for the isolates studied are presented in Table 2.

The list of resistance genes revealed was the same for all isolates, except for the *sul1* gene, providing resistance to sulfonamides, and thus isolate names are not presented in the table below.

All resistance genes were located on the chromosomes.

However, *sul1* was detected in CriePir331 and CriePir332 hybrid assemblies, but not in the short-read assemblies of these isolates. Given that all four isolates are highly likely to constitute a single strain based on genomic data, as will be described below, the absence of *sul1* in the genomes of CriePir333 and CriePir345 can be attributed to short-read assembly inaccuracy. We confirmed this by revealing this gene when setting a low coverage cutoff for the searching algorithm (60% instead of default 80% value).

Virulence and heavy metal resistance-associated genes were also completely the same for all isolates. The list and description of gene clusters are presented in Table 3. All genes were located on the chromosomes.

Isolate typing using Pasteur MLST scheme revealed that all isolates belonged to ST2, which constitutes a major part of a global clone 2 being widespread in many regions of the world [9]. Capsular and lipopolysaccharide types were KL33 and OCL1, respectively.

Since the isolates were similar in their resistance profiles and were attributed to the same MLST/KL/OCL types, we performed an additional comparison using the cgMLST scheme including 2390 loci to check whether these four isolates could be attributed to the same clone. The cgMLST profiles were completely the same (see Table S1), and thus we can suggest that CriePir331, CriePir332, CriePir333 and CriePir345 belong to the same MDR clone of *A. baumannii*.

**Figure 1:**
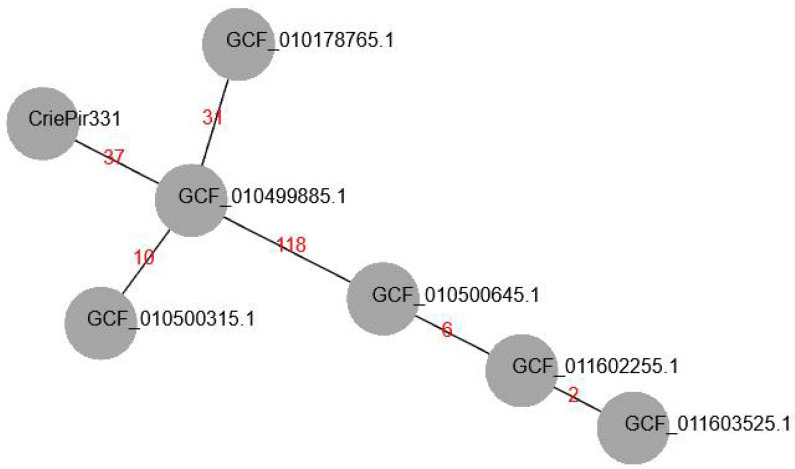
Minimum-spanning tree (MST) of cgMLST allelic profiles build for CriePir331 and ST2 isolates from RefSeq database having the closest profiles to CriePir331. The branch labels indicate the number of differing alleles. All isolates from RefSeq were obtained in the same medical center in India in 2020.

We searched for similar isolates in the RefSeq database (https://www.ncbi.nlm.nih.gov/refseq/, accessed on 20 November 2021) based on cgMLST profiles in order to build a minimum-spanning tree for the nearest matches. At first, we revealed that CriePir87 (GCF_016654375.1, isolated in 2017 in the same hospital) sequenced by us earlier [8] had the same profile as CriePir331 and others except for 22 alleles that were uncalled in either of the two isolates. Thus, CriePir87 was excluded from the tree for obvious reasons. The number of differing alleles between CriePi331 and other reference isolates varied from 37 (GCF_010499885.1) to 133 (GCF_011603525.1). All reference isolates in the tree were obtained in the same Indian medical center in 2020 from blood samples. The minimum-spanning tree for CriePir and reference isolates is given in Figure 1.

Additionally, we searched for plasmid similarities in Genbank, and the closest match was CP035673, which was obtained in the same Indian hospital as the previously mentioned reference isolates. However, the plasmid was longer (16,033 vs. 11,194 in CriePir isolates) and was obtained in 2017. It carried BrnT family toxin, toxin-antitoxin system and TonB receptor. No resistance or virulence genes were annotated on this plasmid similarly to CriePir plasmids.

## 3. Discussion

Our study details the phenotypic and genomic characteristics of clinical *A. baumannii* isolates obtained from COVID-19 patients in the dedicated ICU of a multidisciplinary medical center in Moscow, Russia. All the isolates were resistant to various antibiotics, including imipenem, and dramatically complicated the treatment that ultimately ended in lethal outcomes for three of the four subjects. Carbapenem resistance always draws an attention since these antibiotics, especially meropenem and imipenem, are often used as a second- or even last-line drugs. Unfortunately, carbapenem-resistant isolates are very common in Russia, with imipenem resistance constituting up to 85% [6,8], and the isolates investigated are not an exclusion. From the epidemiological point of view, it is very important to investigate the bacterial coinfections in these cases in order to trace the possible emergence of novel MDR organisms and the spread of antimicrobial resistance within ICU.

Many researchers anticipated the rise of antimicrobial resistance due to the COVID-19 pandemic, and several reports confirming these expectations are already available [10,11,12]. However, other investigations have not revealed significant changes in AMR from 2019 to 2020 in clinical settings, at least as a short-term consequence [13,14]. The reason for such inconsistencies could be that it is rather hard to correctly estimate the actual increase in AMR caused by COVID-19, since the number of patients and duration of their hospital stay have increased significantly, and the unambiguous criteria have not been developed yet. In general, the infections caused by MDR and extensively drug-resistant (XDR) bacteria are associated with dramatically higher morbidity and mortality [15,16,17], and this also holds true for the cases of coinfections in severe COVID-19 patients, for which such coinfections required longer hospitalization and were associated with a higher risk of death [18,19,20]. Some researchers suggested that the empiric use of broad-spectrum antimicrobials for such patients might have led to the selection of MDR organisms [20,21].

It is also important to investigate the nature and origins of bacterial coinfections in ICUs, namely, whether they were caused by the same bacterial clones as those present pre-COVID or by novel strains developed during the pandemic. Such reports are scarce and we have not yet found the published results of such investigations for Russian hospitals.

In our study, the application of the short- and long-read whole genome sequencing (WGS) allowed us to reveal that the same clone of *A. baumannii* has caused multiple cases of bacterial infections in COVID-19 ICU. In addition, this multidrug-resistant clone was virtually the same as the one obtained in this medical center in 2017 in Surgery department [8]. However, such a clone has not been revealed during continuous monitoring in 2018–2019, so we do not have enough data to check whether it was persistent or reintroduced from other healthcare facilities. The closest matches from the RefSeq database in terms of the cgMLST genomic profile were revealed in a single Indian medical center. These reference isolates were obtained in 2020; at the same time, the plasmid obtained in the same facility in 2017 was also very similar to the one possessed by our isolates. Thus, it is possible that such a clone was developed before 2017 and spread across different countries, which is not an unlikely scenario for other isolates belonging to GC2, a dominant international clone of high-risk [9,22].

Our study is limited in a sense that it constitutes only a preliminary report regarding several cases of bacterial coinfections by MDR *A. baumannii* in COVID-19 patients. Currently, we are working on a more comprehensive investigation of such infections involving various bacterial species and spanning several waves of the pandemic. However, we believe that comprehensive genomic analysis of MDR bacterial species that caused infections in COVID-19 ICUs is a pivotal element in studying the changes of antimicrobial resistance profiles caused by the pandemic, especially due to the extensive and, in some cases, inappropriate use of antibiotics. While thorough multicenter genomic epidemiology analysis of such coinfections in Russia has not been proposed yet, to the best of our knowledge, the accumulation of high quality annotated genomes of MDR and pandrug-resistant bacterial species which caused infections in COVID-19 patients will greatly facilitate the development of better antibiotic treatment protocols and infection-preventing measures.

## 4. Materials and Methods

### 4.1. Determination of Antibiotic Susceptibility

Species identification for the isolates studied was conducted by time-of-flight mass spectrometry (MALDI-TOF MS) using the VITEK MS system (bioMerieux, Marcy-l’Étoile, France), and the susceptibility to antimicrobials was determined by the disc diffusion method using the Mueller-Hinton medium (bioMerieux, Marcy-l’Étoile, France) and disks with antibiotics (BioRad, Marnes-la-Coquette, France), and the Minimum Inhibitory Concentration (MIC) method with the VITEK 2 Compact 30 analyzer (bioMerieux, Marcy-l’Étoile, France). Antibiotics panel included the following drugs: ciprofloxacin, gentamicin, imipenem, levofloxacin, netilmicin and trimethoprim/sulfomethoxazole. This is a short version of a panel that was used in ICU reserved for COVID-19 patients due to limited resources’ availability and other technical issues caused by the first wave of pandemic. We used the EUCAST clinical breakpoints, version 11.0 (https://www.eucast.org/clinical_breakpoints/, accessed on 20 December 2020) to interpret the susceptibility/resistance results obtained.

### 4.2. DNA Isolation, Sequencing and Genome Assembly

Four samples were obtained from blood (CriePir331 only, female patient) and the bronchoalveolar lavage fluid of four patients (3 males and 1 female) in ICU reserved for the subjects with confirmed COVID-19 disease at a multidisciplinary federal medical center in Moscow, Russia during June 2020. The age of the patients involved in this study ranged from 66 to 78 years. The samples studied were randomly chosen from the whole set of samples used for antibiotic-susceptibility testing. MDR *A. baumannii* isolates were chosen for this study. No additional bacterial species were revealed in the patient samples selected for this investigation.

Two representative isolates (CriePir331 and CriePir332) were selected for long-read sequencing that allowed us to obtain the precise genome and plasmid structures, as well as to verify the locations of antibiotic resistance and virulence determinants, and to obtain complete cgMLST profiles for the selected isolates.

Genomic DNA was isolated using a DNeasy Blood and Tissue kit (Qiagen, Hilden, Germany), while a Nextera™ DNA Sample Prep Kit (Illumina^®^, San Diego, CA, USA) was applied for paired-end library preparation and the WGS of the isolates on Illumina^®^ Hiseq 2500 platform (Illumina^®^, San Diego, CA, USA). The same genomic DNA was used to produce the libraries for the Oxford Nanopore MinION sequencing system (Oxford Nanopore Technologies, Oxford, UK) with the Rapid Barcoding Sequencing kit SQK-RBK004 (Oxford Nanopore Technologies, Oxford, UK). The amount of initial DNA was 400 ng for each sample. The libraries were prepared according to the manufacturer’s protocols, and were sequenced on R9 SpotON flow cell with a standard 24 h sequencing protocol using the MinKNOW software version 21.06.13 (Oxford Nanopore Technologies, Oxford, UK).

Base calling of the raw MinION data was performed using Guppy Basecalling Software version 4.4.1 (Oxford Nanopore Technologies, Oxford, UK), and demultiplexing was performed using Guppy barcoding software version 4.4.1 (Oxford Nanopore Technologies, Oxford, UK). Hybrid short- and long-read assemblies were obtained using Unicycler version 0.4.9 (normal mode) [23].

Genome assemblies were uploaded to NCBI Genbank under the following accession numbers: JAJTJI000000000 (CriePir331), JAJTJH000000000 (CriePir332), JAJTJG000000000 (CriePir333), JAJTJF000000000 (CriePir345).

### 4.3. Data Processing

The genomes that were assembled were processed using the custom software pipeline described earlier [24]. We used the Resfinder 4.0 database for antimicrobial gene detection (https://cge.cbs.dtu.dk/services/ResFinder/, accessed on 20 December 2021). Virulence factors were revealed by searching in VFDB (http://www.mgc.ac.cn/VFs/main.htm, accessed on 20 December 2021).

Isolate typing was first performed by MLST using the Pasteur scheme [25]. Additional typing was completed using the capsule synthesis loci (K-loci) [26] and lipooligosaccharide outer core loci (OCL) [27]. These data form the basis for the classification of *A. baumannii* isolates that is important for their identification and epidemiological surveillance [28]. Detection of cgMLST profiles was performed using MentaList (https://github.com/WGS-TB/MentaLiST, accessed on 10 February 2022, version 0.2.4, default parameters) [29] using the scheme developed by Higgins et al. [30].

## 5. Conclusions

Here, we presented a comprehensive genomic analysis of four multidrug-resistant *A. baumannii* isolates obtained from the patients of a dedicated COVID-19 ICU. By using the advances of short- and long-read whole genome sequencing, we were able to characterize the factors involved in antibiotic resistance and the virulence mechanisms of these isolates, as well as to determine the plasmid structures. A genomic epidemiology analysis based on the cgMLST profiles allowed us to reveal the absolute similarity between the isolates studied and the one previously obtained in the same hospital. We believe that our data will contribute to the investigation of the possible changes in antibiotic resistance due to extensive drug use during the COVID-19 pandemic, which, in turn, could lead to the development of better treatment protocols for infections caused by *A. baumannii* in clinical settings.

## Figures and Tables

**Table 1:** Antibiotic resistance phenotype and metadata for *A. baumannii* isolates.

Isolate	Patient Gender	Patient Age	Isolation Source	Ciprofloxacin	Gentamicin	Imipenem	Levofloxacin	Netilmicin	Tmp/Smz ^1^
CriePir331	Female	75	Blood	R	R	R	R	R	R
CriePir332	Male	78	BAL ^2^	R	R	R	R	R	R
CriePir333	Male	76	BAL	R	R	R	R	R	R
CriePir345	Male	66	BAL	R	R	R	R	R	R

^1^ Tmp/Smz—trimethoprim/sulfamethoxazole, ^2^ BAL—bronchoalveolar lavage.

**Table 2:** Antibiotic resistance genotype of *A. baumannii* isolates.

Gene	Function	Affected Antimicrobials
*arr-2*	integron-encoded ribosyltransferase	Rifampicin
*ant(3″)-Ia*	aminoglycoside nucleotidyltransferase	Aminoglycosides
*aph(3″)-Ia*	aminoglycoside phosphotransferase	Aminoglycosides
*aph(3″)-Ib*	aminoglycoside phosphotransferase	Aminoglycosides
*aph(6)-Id*	aminoglycoside phosphotransferase	Aminoglycosides
*armA*	16S rRNA methyltransferase	Aminoglycosides
*blaADC-30*	intrinsic ADC beta-lactamase and cephalosporinase of *A. baumannii*	Cephalosporines
*blaOXA-23*	carbapenemase	Carbapenems
*blaOXA-66*	intrinsic OXA-51-like β-lactamase of *A. baumannii*	Cephalosporines
*blaPER-7*	extended-spectrum β-*lactamase*	Penicillins, Cephalosporines
*catB8*	chloramphenicol acetyltransferase	Chloramphenicol
*cmlA5*	chloramphenicol exporter	Chloramphenicol
*mph(E)*	macrolide phosphotransferase and resistance gene	Macrolides
*msr(E)*	ABC-F subfamily protein	Macrolides
*qacE*	resistance gene conferring resistance to antiseptics	Antiseptics
*sul1*, *sul2*	sulfonamide resistant dihydropteroate synthase	Sulfonamides
*adeABCRS*	multidrug efflux complex and its regulators	β-lactams
*adeFGHL*	multidrug efflux complex and its regulators	Fluoroquinolones
*ompA*	porin, permeability defects	All

**Table 3:** Virulence and heavy metal resistance-associated genes of *A. baumannii* isolates.

Gene Cluster	Function
*abaI*	virulence, motility, conjugation, biofilm formation and host-pathogen interactions
*bap*	biofilm formation
*barAB*	siderophore efflux system
*basABCDFGHJ*	iron acquisition system and acinetobactin functioning
*bauABCDEF*	iron acquisition system and acinetobactin functioning
*bfmRS*	quorum sensing-regulated two-component system involved in biofilm formation
*csuABCDE*, *csuA/B*	biofilm formation
*entE*	enterobactin biosynthesis (siderophore)
*htpB*	heat shock protein
*katA*	oxidative stress resistance
*pgaABCD*	biofilm formation
*pilT*	twitching motility and evasion of host immune system
*plc*, *plcD*	lipolytic activity for iron acquisition

## Data Availability

The assembled genome sequences for all isolates were uploaded to the NCBI Genbank under the project number PRJNA789572. Genomic sequences are available under the following accession numbers: JAJTJI000000000 (CriePir331), JAJTJH000000000 (CriePir332), JAJTJG000000000 (CriePir333), JAJTJF000000000 (CriePir345).

## Supplementary Materials

The following supporting information can be downloaded at: https://www.mdpi.com/article/10.3390/antibiotics11030346/s1, Table S1. cgMLST profiles of *A. baumannii* isolates studied and reference isolates from RefSeq database.
